# Death trends based on autopsy data compared to the beginning of the coronavirus pandemic in Brazil

**DOI:** 10.1590/1414-431X202010766

**Published:** 2021-02-12

**Authors:** G.A.A. Gimenez, P.K. Zilli, L.F.F. Silva, C.A. Pasqualucci, A.B. Campo, C.K. Suemoto

**Affiliations:** 1 Programa de Patofisiologia, Faculdade de Medicina, Universidade de São Paulo, São Paulo, SP, Brasil; 2 Pesquisador independente, São Paulo, SP, Brasil; 3 Departamento de Patologia, Faculdade de Medicina, Universidade de São Paulo, São Paulo, SP, Brasil; 4 Departamento de Engenharia Elétrica, Instituto Federal de São Paulo, São Paulo, SP, Brasil; 5 Divisão de Geriatria, Faculdade de Medicina, Universidade de São Paulo, São Paulo, SP, Brasil

**Keywords:** Coronavirus, Covid-19, Time-series, Artificial intelligence, Statistics

## Abstract

The novel Coronavirus disease (COVID-19) is responsible for thousands of deaths worldwide, especially in Brazil, currently one of the leading countries in number of infections and deaths. The beginning of the COVID-19 epidemic in Brazil is uncertain due to the low number of tests done in the country. The excess number of deaths can suggest the beginning of the pandemic in this context. In this article, we used an autoregressive integrated moving average (ARIMA) model to investigate possible excesses in the number of deaths processed by the São Paulo Autopsy Service according to different causes of deaths: all-cause, cardiovascular, and pulmonary causes. We calculated the expected number of deaths using data from 2019 to 2020 (n=17,011), and investigated different seasonal patterns using harmonic dynamic regression with Fourier terms with residuals modeled by an ARIMA method. We did not find any abnormalities in the predicted number of deaths and the real values in the first months of 2020. We found an increase in the number of deaths only by March 20, 2020, right after the first COVID-19 confirmed case in the city of São Paulo, which occurred on March 16, 2020.

## Introduction

The world is facing a pandemic due to a novel coronavirus (Sars-Cov-2) since January 30, 2020, when the World Health Organization (WHO) declared the outbreak a public health emergency of international concern (1). The first reported case of the new coronavirus disease, named COVID-19, was in early December in Wuhan, China (2), and as of August 11, 2020, the disease has killed more than 732,000 people and infected almost 20 million worldwide (1). As of December 3, 2020, Brazil was the second country with most deaths and infections due to COVID-19 with more than 3 million reported cases and 101,049 deaths, only behind the United States of America that has almost 5 million reported cases and 161,547 deaths (1). The first death by COVID-19 in Brazil occurred in the city of São Paulo on March 16, 2020.

Most initial cases of COVID-19 in western countries were of individuals returning from China (3) and Italy (4), but many countries still have an issue for establishing when the first case of local transmission occurred. This is a consequence of many problems; one of them is the existence of a fraction of asymptomatic individuals (due to mild or lack of symptoms) that facilitated the dissemination of the coronavirus before its detection by the health system (5), combined with insufficient testing, which limited the capacity to count the number of infections and, consequently, establish the real timeline of the coronavirus infection. On the other hand, the excess mortality rate can provide valuable information about the dynamics of the pandemic (6). Recently, this approach was used in several studies. Li et al. established that a great number of infections in China happened long before social restrictions and control policies took place, revealing a large number of undocumented cases (5). Also, Delatorre et al. (7) confirmed such findings for multiple countries, including the USA, UK, and Italy. For Brazil, they found that COVID-19 was already circulating in the Brazilian population in early February (between January 31st and February 7th), 2020.

Following the trend of using the excess number of deaths, many time-series models can be used to forecast new deaths and understand the coronavirus dynamics. One of them is the auto regressive integrated moving average (ARIMA) model. The ARIMA model, or Box-Jenkins model, was developed in 1976 by George Box and Gwilym Jenkins, who introduced the concept of transforming non-stationary data into stationary (8). Data is considered stationary when its parameters, mean and variance, do not depend on time (i.e., are constant throughout time) (9). The transformation introduced by Box and Jenkins is called seasonal differencing and consists of computing the differences between consecutive observations.

Using the excess number of deaths is preferred over the number of notified infected cases, mainly because of the small number of tested people leading to sub-notification in the number of cases (7). Therefore, the objective of this study was to explore the number of autopsies performed at the São Paulo Autopsy Service (SPAS) to find a possible excess in the number of deaths before the first official death by Sars-CoV-2 in the city of São Paulo. This analysis can help us to establish if deaths related to the coronavirus occurred previous to March 16, 2020, implying a possible early community transmission of the virus and the spread of the disease in Brazil for some time before the first official case.

## Material and Methods

### Participants

São Paulo is the largest city in Brazil, with an estimated population near 12 million in 2019 (10). Data on mortality were collected from the SPAS, which since 1931 is responsible for clarifying the cause of non-traumatic deaths that were due to ill-defined disease or of people without medical assistance in the municipality of São Paulo. SPAS performs more than 14,000 full-body autopsies yearly, accounting for almost 20% of the autopsies in the city of São Paulo (11).

Socioeconomic and clinical data are collected by the SPAS staff from the next of kin. The causes of death determined with the information from the autopsy examination were coded according to the 10th revision of the International Statistical Classification of Diseases and Related Health Problems (ICD-10) (12).

The SPAS stopped the conventional autopsy procedure on March 21st, 2020 following São Paulo State regulations based on the high risk of staff contamination during the COVID-19 pandemic, which caused an interruption in data collection. Therefore, this study included death data from January 1st, 2019 to March 20th, 2020. We excluded individuals younger than 18 years (n=1,641), cases for which the date of autopsy was after March 20, 2020, and a few cases with data processing errors (n=63). Family members signed an informed consent after a SPAS employee explained the autopsy procedure and the possible participation in a research project.

### Autopsy evaluation

Individuals who die from an undefined cause are referred to the SPAS, where a full-body autopsy is performed to determine the cause of death. Upon the arrival of the deceased body at the SPAS, the next of kin is requested to answer a few questions regarding the moment of death, previous diseases, and lifestyle factors. The average postmortem interval between death and autopsy exam in our sample was 24.77±5.64 h.

Autopsies are performed by certified pathologists that examine major organs macroscopically. When necessary, tissue samples are collected for additional microscopic analysis. Afterward, the pathologist determines the cause of death based on the findings from the autopsy exam. The basic and immediate causes of death are determined, as well as additional pathologies that could have contributed to the death. For the all-cause mortality analysis, all ICD-10s (12) are included. Also, specific-cause mortality is determined by selecting ICD-10s that were possibly related to the coronavirus: pneumonia (J18), respiratory-related (ICD-10s in Table 1), and coronary artery disease (I21 and I25) cases (13,14).

**Table 1:** Table 1. Selected International Classification of Diseases (ICD)-10 of respiratory diseases possibly related to coronavirus disease (COVID-19).

ICDs	Disease
J00	Acute nasopharyngitis
J06	Acute upper respiratory infections of multiple and unspecified sites
J09	Influenza due to identified zoonotic or pandemic influenza virus
J10	Influenza due to identified seasonal influenza virus
J12	Viral pneumonia, not elsewhere classified
J13	Pneumonia due to *Streptococcus pneumoniae*
J14	Pneumonia due to *Haemophilus influenzae*
J15	Bacterial pneumonia, not elsewhere classified
J16	Pneumonia due to other infectious organisms, not elsewhere classified
J17	Pneumonia in diseases classified elsewhere
J18	Pneumonia, organism unspecified
J20	Acute bronchitis
J21	Acute bronchiolitis
J22	Unspecified acute lower respiratory infection
J40	Bronchitis, not specified as acute or chronic
J80	Adult respiratory distress syndrome
J96	Respiratory failure, not elsewhere classified

### Model training and testing

Because the main focus of this study was to understand the death pattern at the beginning of the pandemic in São Paulo, we divided the data into two sets: training and test. The model was trained using data from the training set and the test set was later used to estimate the model performance because we were interested in estimating the model accuracy on genuine forecasts, which refers to data that was not used when fitting the model. To validate and choose our models, we performed the classical holdout technique splitting our data following the 80-20 proportion: the training set consisted of death records from January 1, 2019 to October 19, 2019, and the test set from October 20, 2019 to January 1, 2020. We chose the best model considering its performance on the test set. Thereafter, we forecasted daily aggregated values from January 1, 2020 to March 20, 2020, and looked for abnormalities comparing the forecasts with the real values from the series. We chose the best model considering its performance on the test set.

### Model fitting

The ARIMA model can be decomposed into two models: 1) autoregression (AR): in a linear regression model, a target variable is forecasted using a linear combination of covariates. In an AR, we forecast the target variable using a linear combination of past values of the target variable (15). The term *p* indicates the order of the AR model or the number of past values that are used; 2) moving average (MA): instead of using past values of the target variable, the MA model uses past forecast errors. In other words, the regression accounts for the current value of the series against a Gaussian white noise distribution (15). The term *m* indicates the order of the MA model or the number of error components used.

To incorporate both of these models, Box and Jenkins proposed a methodology that used stationary transformations to integrate both models. Thus, in the integration component in the ARIMA model, the term *i* indicates the number of differentiations performed to transform the non-stationary series into a stationary one. The task of estimating the parameters of the ARIMA or Box-Jenkins model can sometimes be subjective and difficult. To solve this, we first used the Hyndman-Khandakar algorithm (16) to estimate the order of the model and we later performed a residual analysis to validate the model chosen by the algorithm.

The default behavior for the algorithm can be summarized in the following steps: a) because ARIMA modeling requires stationary time-series, the number of differences (i.e., obtained subtracting consecutive observations) is determined using repeated Kwiatkowski-Phillips-Schmidt-Shin (KPSS) tests; b) the values of *p* (the autoregressive component of the model) and *q* (the moving-average component of the model) are chosen by minimizing the small-sample corrected Akaike information criterion (AICc) after differencing the data, which includes a penalty term for complex models. The algorithm uses a stepwise search to transverse the solution space to find the optimal *p* and *q* parameters. This step consists of searching for the best model defined by the lowest AICc among the simple ARIMA models and making variations to the best model until no lower AICc can be found. To perform the residual analysis, checking if the model had adequately captured all the information in the data and can be used for prediction, we visually checked for correlations in the residuals using autocorrelation plots (ACF) and partial autocorrelation plots (PCF). We have also performed portmanteau tests to assert that there was no correlation using the Ljung-Box test. Furthermore, we calculated the mean value for the residuals, looking for a zero mean value, to assert that the model did not have any bias.

There are some challenges when dealing with long daily time-series data, such as dealing with multiple seasonal patterns and long-term seasonal patterns. To account for these effects, we tested two approaches: a) pure ARIMA modeling: components were estimated using the previously mentioned Hyndman-Khandakar algorithm; b) ARIMA modeling with time series decomposition: we performed decomposition of the series with the Seasonal-trend decomposition procedure with loess (17) and used an ARIMA modeling in the seasonal-adjusted component. The ARIMA estimated components were obtained using the Hyndman-Khandakar algorithm.

When evaluating these two approaches, we selected the best model in each one and compared the performance on the test set. The winning model was the one that had the best performance (i.e., the smaller error). When the errors were too close, we chose the model with a smaller number of degrees of freedom (i.e., the simplest model).

### Statistical analysis

To estimate the accuracy of the model in genuine forecasts, we used the mean absolute error [*MAE* = *mean*(|*e*t|)], a scale-dependent error that refers to the differences between the real and the forecasted death count. This was calculated using the forecasts obtained in the test set.

We used mean and standard deviation to describe continuous variables and percentages for categorical ones. Characteristics of individuals were compared using unpaired Student's *t*-test and chi-square tests, when appropriate. To calculate the annual mortality rates, we performed a population correction using the estimated total number of inhabitants in the city of São Paulo according to the State System of Data Analysis Foundation (10). The alpha level was set to 0.05. Data analysis was performed using the R 3.6.3 software (18).

## Results

### Characteristics of the sample

In total, there were 17,011 autopsies conducted at the SPAS from 2019 to 2020 (54.5% men) with an average age of 68.7±15.6 years. Table 2 describes the baseline characteristics of the training, test, and 2020 datasets. Individuals were older adults, predominantly male, and white with elementary education. Diabetes was one of the most common risk factors followed by smoking habit. In our sample, coronary artery disease (ICD I21 and I25) was one of the most common causes of death comprising almost 25% of the cases for all three datasets (training, test, and 2020 dataset).

**Table 2:** Baseline characteristics of the study participants (n=17,011).

Characteristic	Training (n=11,313)	Test (n=2,693)	2020 dataset (n=3,005)	P^a^	P^b^
Age (years), mean±SD	68.74±15.51	68.72±15.56	68.51±15.72	0.74^d^	0.48^d^
Male (%)	54.94	54.25	53.75	0.47^c^	0.18^c^
Education (%)				0.002^c^	<0.001^c^
Illiterate	11.98	11.30	9.21		
Elementary	41.89	40.59	43.11		
Primary	21.48	21.90	22.25		
High School	19.11	19.44	19.52		
College or higher	5.53	6.77	5.92		
Race (%)				0.06^c^	0.52^c^
White	70.73	71.43	70.99		
Brown	21.14	19.68	20.74		
Black	7.19	7.59	7.54		
Other	0.95	1.30	0.73		
Comorbidities (%)					
Diabetes	31.08	32.68	31.08	0.39^c^	0.42^c^
Asthma	4.84	4.57	4.49	0.34^c^	0.24^c^
Bronchitis	5.71	5.90	5.39	0.86^c^	0.28^c^
AIDS	0.81	0.78	0.47	0.73^c^	0.02^c^
Epilepsy	2.22	2.56	1.73	0.73^c^	0.04^c^
Smoker	26.05	25.92	24.93	0.29^c^	0.02^c^
Cause of death (%)				<0.001^c^	<0.001^c^
Coronary artery disease	23.54	21.43	23.03		
Pneumonia	12.07	12.96	11.81		
Respiratory diseases	14.14	13.29	12.25		
Other	50.24	52.32	52.91		

^a^Comparison between training and test sets; ^b^Comparison between training and 2020 dataset; ^c^Chi-squared test; ^d^unpaired *t*-test.

The cause of death distribution was statistically different for all three datasets, with an increase in the ‘Other' category and a decrease in most of the selected causes of death. When comparing our training and test datasets, we only found significantly different frequencies for education. For the training set and the 2020 dataset, we also found different distributions for education and for AIDS, epilepsy, and smoking habits (Table 2).

### Temporal analysis

The average monthly mortality rate during the period was 110.17 per 100,000 individuals. In the period of one year (January 2019 to January 2020), the monthly all-cause mortality rate increased from 8.84/100,000 in 2019 to 9.41/100,000 in 2020 (a 5.99% increase).

For coronary artery disease and pneumonia, we found evidence of a 7-day seasonal pattern, but for respiratory-related issues the seasonal pattern was less identifiable resembling a 6-day seasonal pattern; for the all-cause mortality, we found evidence of a 365-day seasonal shape pattern.

When looking at daily data, the average number of daily deaths during the period (2019-2020) was 38.22±7.29 with 4.65±2.88 pneumonia deaths, 5.22±3.02 deaths due to respiratory diseases, and 8.83±4.25 by coronary artery disease.

**Figure 1:**
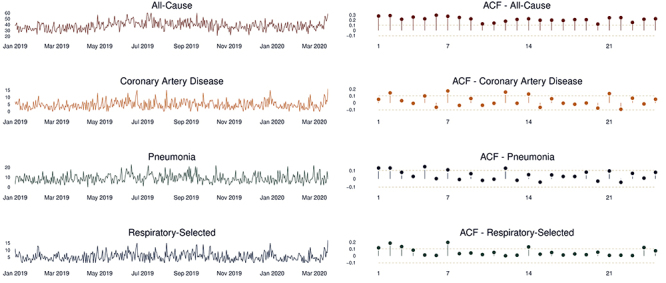
Time-series and autocorrelation function (ACF) plot for all-cause mortality and specific causes (coronary artery disease, respiratory diseases, and pneumonia).

Figure 1 shows the time-series plots and the autocorrelation functions (showing the seasonal patterns described above) for all-cause, pneumonia, respiratory-related, and coronary artery disease death cases.

### Model fitting and forecasting

For all subseries, all-cause and specific causes of death (coronary artery disease, respiratory disease, and pneumonia), the best model was the pure ARIMA model, giving the best out-of-sample mean absolute error (MAE) metrics. The results of portmanteau tests on the residuals showed that the pure ARIMA models had no correlations left on the residuals and, because of that, these were suitable for making predictions (Table 3).

**Table 3:** Comparison of various autoregressive integrated moving average (ARIMA) methods and modeling approaches for all-cause and specific causes of deaths.

Series/Models	MAE	Residual analysis*	MAE^†^
All-cause			
ARIMA(0,1,1)	5.3247	Yes	4.7015
STL + ARIMA(0,1,1)	4.8051	No	4.7548
Pneumonia			
ARIMA(2,1,4)	2.1547	Yes	2.1589
STL + ARIMA(1,1,1)	1.9049	No	2.4459
Coronary artery disease			
ARIMA(2,1,3)	3.2742	Yes	2.9717
STL + ARIMA(2,1,2)	2.8603	No	3.3132
Respiratory diseases			
ARIMA(4,1,2)	2.3458	Yes	2.2681
STL + ARIMA(1,1,2)	2.1199	No	2.6582

MAE: mean absolute error for the in-sample forecast. *P<0.01 for the portmanteau test performed on residuals. ^†^MAE for out-of-sample forecast.

**Figure 2:**
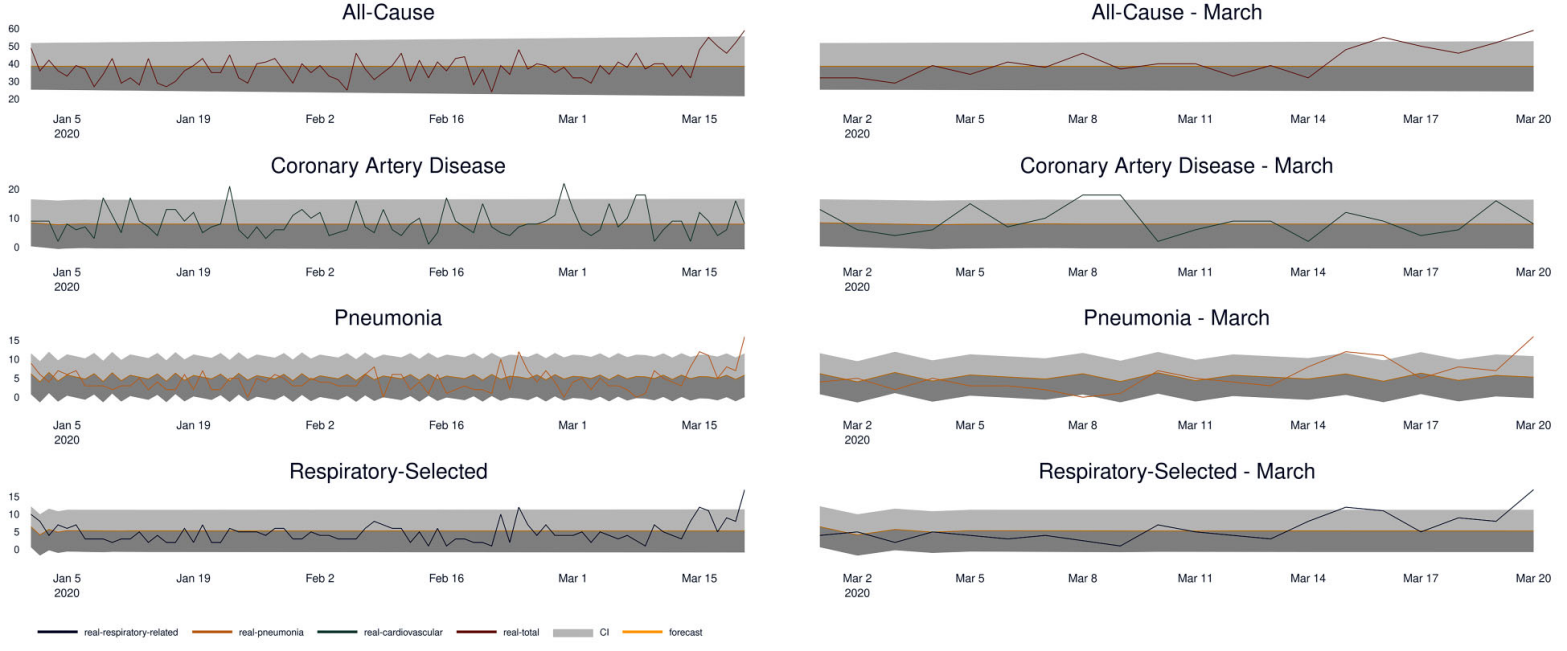
Left panel: Expected (forecast) and observed (real) number of deaths from January to March 2020. Right panel: Expected (forecast) and observed (real) number of deaths in March 2020.

The model for coronary artery disease indicated an excess of deaths for four dates in 2020: January 23rd, February 29th, March 8th, and March 9th. For pneumonia, the chosen model only indicated an excess of deaths on 2 dates: March 16th and March 20th. For respiratory diseases, the chosen model also only indicated an excess of deaths on 2 dates: March 16th and March 20th. Finally, the all-cause mortality series model revealed an excess of deaths on March 16th and March 20th. Figure 2 shows the forecast for each one of the series and their confidence intervals for the period of January 1 to March 20, 2020.

## Discussion

In this study, we investigated whether an excess number of deaths occurred in the SPAS during the first trimester of 2020 using forecasts from a time-series model trained on daily data from January 2019 to mid-October 2019. We focused on the all-cause mortality and selected coronary artery disease and respiratory ICDs related to COVID-19 according to multiple reports (13,14).

For the all-cause mortality, we only found two dates with an excess of deaths (with 3 and 6 more deaths than expected for March 16th and 20th, respectively) from our model forecasted from previously observed values. In São Paulo, the health crisis due to the pandemic started around the end of April, when the ICU hospitalization rate for COVID-19 reached 67% and the number of deaths due to the virus reached 1,124 confirmed cases and 1,603 suspected cases (19). Therefore, we did not expect to find an excess in the number of deaths for the all-cause mortality related to the pandemic.

InfoGripe, a system from Fundação Oswaldo Cruz that monitors cases of hospitalization and presents alert levels for cases of severe acute respiratory syndrome (SARS) in Brazil, recorded a ten-fold increase in the historical average of hospitalizations on April 31, 2020 (20), that is, after the notification of the first case in Brazil on February 25, 2020. Because of that, we expected to find possible abnormalities or excess deaths due to pulmonary causes, especially for pneumonia and SARS. Nevertheless, for pneumonia, we only found an excess in the number of deaths on March 15th (1 more than expected) and March 20th (5 more than expected), the latter being the last day of our daily time-series and when the city of São Paulo started to apply public policies to contain the pandemic because of the first death on the 17th of March. The increase above the forecasted confidence intervals matched the overall health crisis that started in São Paulo, with social distancing policies being enforced to prevent more infections. Furthermore, we found an excess of deaths on the same dates due to respiratory disease, mainly driven by pneumonia. Finally, for coronary artery disease death cases, we found a few days with an excess in the predicted number of deaths by our model, but they lacked a constant increasing pattern that could be related to the COVID-19 pandemic.

Although we expected to see an abnormal increase in deaths on these series, especially for pneumonia and the respiratory ICDs, our results suggested that only in mid-March did deaths begin to increase above the confidence intervals of the models. This did not contradict evidence that COVID-19 reached Brazil in early-February, as reported in several studies (7,21), but suggested that the mortality dynamics for the virus in Brazil were slower than other Western countries probably because of the warm months in the first trimester, which was also reported previously (22).

There are some limitations to this study. First, our data (truncated at March 20, 2020) correspond to the beginning of the pandemic in Brazil, making it hard to make conclusions from March onwards. Additional observations after this date would allow more significant findings regarding the COVID-19 evolution in São Paulo. Second, due to the context of the pandemic, many autopsy protocols suffered changes to reduce contamination risks. In São Paulo, the first official resolution by the Mayor’s office of the City of São Paulo took place on March 20, 2020 (23), establishing rules for the investigation of death cases including indirect and verbal autopsy procedures for suspected cases and expansion in the use of less invasive tools. It is worth mentioning that due to this resolution the SPAS stopped the conventional autopsy procedure on March 21, 2020. Third, the ARIMA modeling approach only accounts for one type of seasonality, which means that other seasonality patterns may not have been incorporated by the model and that could affect its performance since we are not using any covariates.

The analysis conducted in this article provides interesting insights into the dynamics of the pandemic during the first trimester of 2020, especially because data comes from a heterogeneous source, representative of the city of São Paulo (the largest city in Brazil). Also, because data come from autopsy reports, the cause of death is much more reliable than clinical data. Our study suggests that mortality and morbidity rates in warm regions had lower growth than in cold regions.

Future work could use hierarchical time-series modeling to accumulate effects from different death causes or using covariates accounting for different events that could have an impact on mortality (like the Brazilian Carnival). In conclusion, our findings are in line with official information that reported that the first death due to the coronavirus in Brazil occurred in mid-March since we did not observe an excess in the number of deaths before this period.
